# Factors Associated With Decent Work Perception Among Chinese Medical Nursing Assistants: A Cross‐Sectional Study With Exploratory Mediation Analysis

**DOI:** 10.1002/brb3.71752

**Published:** 2026-10-13

**Authors:** Jinhong Lu, Juan Liu, Huina Mao, Guizhi Luo

**Affiliations:** ^1^ Department of General Surgery, Zhujiang Hospital Southern Medical University Guangzhou China; ^2^ Department of Thoracic Surgery, Zhujiang Hospital Southern Medical University Guangzhou China; ^3^ Nursing Department, Zhujiang Hospital Southern Medical University Guangzhou China

**Keywords:** China, cross‐sectional study, decent work perception, medical nursing assistants, occupational burnout, psychological capital

## Abstract

**Background:**

Medical nursing assistants are non‐professional auxiliary care workers who provide daily living care and assist with basic activities under the guidance of healthcare professionals in China. Guided by the Job Demands‐Resources (JD‐R) model, this study examined how job demands, job resources, and personal resources were associated with decent work perception and whether occupational burnout statistically accounted for part of the psychological capital‐decent work perception association.

**Methods:**

A cross‐sectional convenience sample of 846 medical nursing assistants was recruited from 21 general hospitals in Guangdong Province, China, in December 2025. The primary regression used variables selected through univariate screening at two‐tailed *p* < 0.05, while JD‐R categories were prespecified for conceptual interpretation. Random forest reproducibility was evaluated through nested five‐fold cross‐validation, explicit hyperparameter tuning, held‐out repeated permutation importance, and conditional permutation for correlated predictors.

**Results:**

The primary regression explained 42.8% of the variance and achieved a five‐fold cross‐validated R2 of 0.414; the random forest nested cross‐validated R2 was 0.393. Psychological capital, occupational burnout, and innovation climate remained the leading random‐forest predictors. CR2 standard errors with Satterthwaite degrees of freedom supported six of the eight primary adjusted associations; daily working hours and insurance were attenuated to *p* = 0.059 and *p* = 0.057, respectively.

**Conclusion:**

Decent work perception was associated with personal resources, job demands, and organizational resources. The cross‐sectional indirect association through occupational burnout was small and does not establish temporal causality.

## Graphical abstract

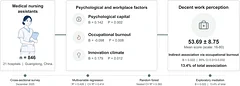

## 1 Introduction

Decent work is a normative concept encompassing fair income, security, dignity, opportunities for development, and social recognition, whereas decent work perception refers to workers' subjective evaluation of whether their employment meets these standards (BowenXue et al. 2024, Xue et al. 2025, Zheng et al. 2025). In this manuscript, decent work perception is used consistently for the construct measured by the Decent Work Perception Scale. In China, medical nursing assistants are auxiliary service personnel rather than registered healthcare professionals. They provide daily living care and assist with activities under professional guidance, but they are not permitted to perform professional nursing procedures. They can be hired directly by the hospital or supplied through third‐party labor organizations. They are required by national guidelines to have specific training and to be managed in a certain way (Ministry of Human Resources, and Social Security, et al. 2019, Sun et al. 2024). Previous studies have shown that medical nursing assistants may have limited professional recognition, job insecurity, strenuous workloads, and inadequate rewards (Hult et al. 2025, Sun et al. 2024). These occupational characteristics make the perception of decent work for medical nursing assistants closely related to the stability and well‐being of the workforce.

Psychological capital is a positive personal resource that is open to development, and consists of self‐efficacy, hope, optimism, and resilience (Luthans and Youssef‐Morgan 2017). These dimensions may foster confidence to accomplish difficult tasks, goal‐directed planning, positive expectations, and recovery from workplace adversity. Previous studies have found a link between psychological capital and reduced occupational burnout and more positive work‐related outcomes (Orgambídez et al. 2025, Tang et al. 2023). Occupational burnout is a syndrome related to work and consists of three dimensions: emotional exhaustion, depersonalization or cynicism, and reduced personal accomplishment (Maslach and Jackson 1981, Maslach et al. 2001). It is conceptually distinct from general fatigue. Therefore, the term occupational burnout is used consistently unless fatigue itself was measured. Studies in nursing populations have reported associations among decent work perception, occupational burnout, work engagement, and turnover intention (BowenXue et al. 2024, Xue et al. 2024). However, relatively few studies have examined whether occupational burnout statistically explains part of the cross‐sectional association between psychological capital and decent work perception specifically among Chinese medical nursing assistants.

The Job Demands‐Resources (JD‐R) model provides the overarching theoretical framework for the present study (Bakker and Demerouti 2017). The JD‐R framework was used before result interpretation to define conceptual roles: daily working hours as a job demand; leadership concern, innovation climate, workplace counseling or training opportunities, and insurance as job or organizational resources; psychological capital as a personal resource; and education and other demographic or employment variables as contextual characteristics. This conceptual organization did not determine statistical entry into the primary regression. Regression terms were selected through data‐driven univariate screening and model building. The first‐stage regression therefore mapped the adjusted associations of the selected variables within their prespecified JD‐R roles, whereas the second‐stage mediation model tested the narrower health‐impairment specification linking psychological capital, occupational burnout, and decent work perception.

The ordering of psychological capital, occupational burnout, and decent work perception was selected a priori on theoretical grounds. First, psychological capital is a relatively developable but enduring positive psychological resource, including self‐efficacy, hope, optimism, and resilience (Luthans and Youssef‐Morgan 2017). It is therefore more consistent with an antecedent personal resource than with a transient work‐state outcome. Second, under the JD‐R health‐impairment pathway, insufficient resources and persistent demands contribute to resource depletion and burnout, which then shapes work‐related evaluations and outcomes (Bakker and Demerouti 2017). Third, conservation of resources theory proposes that individuals strive to obtain and protect resources, and resource loss is central to stress responses (Hobfoll 1989). Occupational burnout can therefore be conceptualized as a resource‐depletion state through which psychological capital is linked to workers' appraisal of whether work is secure, respected, rewarding, and developmentally meaningful. ILO decent work theory was used to define the dependent construct, decent work perception, whereas JD‐R, conservation of resources theory, and psychological capital theory together guided the explanatory ordering. We acknowledge that reverse or reciprocal pathways are possible in cross‐sectional data, and Ferraro et al. have examined decent work as a predictor of psychological capital (Ferraro et al. 2018). However, those alternative models address a different theoretical question and were not selected as the main model because our primary focus was the resource‐protection pathway from personal resource to burnout and subsequent decent work perception.

The primary aim of this study was to identify JD‐R‐classified personal, work‐related, and organizational variables independently associated with decent work perception among Chinese medical nursing assistants. The secondary aim was to test a theory‐driven statistical indirect pathway in which occupational burnout accounted for part of the association between psychological capital and decent work perception. This design allowed the study to first establish the broader JD‐R‐based correlates of decent work perception and then examine a focused psychological resource‐depletion pathway.

## 2 Methods

### 2.1 Study Population and Sampling

This study was a cross‐sectional survey of 846 medical nursing assistants recruited by convenience sampling in 21 general hospitals, Guangdong Province, China, in December 2025. Medical nursing assistants were defined as auxiliary workers who provided daily living care and assisted with basic activities under the guidance of healthcare professionals. They were distinct from registered nurses and could be employed by hospitals or third‐party management organizations. Inclusion criteria were current work in this position and age 18 years or older. Exclusion criteria were absence from duty because of vacation, external assignment, illness, or other reasons, or a stated plan to resign in the near future. The minimum sample size was calculated for 25 candidate independent variables with a 5–10 participants per variable ratio (Christopher Westland 2010), and accounting for incomplete questionnaires. The final sample of 846 exceeded this minimum. The study was approved by the institutional ethics committee (2025‐KY‐478‐01), and ethical principles were followed.

### 2.2 Measures

#### 2.2.1 General Information Survey Form

Demographic, employment and organizational information was collected through a researcher‐designed form including the participant's medical institution, age, sex, marital status, education, income, professional rank, work experience, employment purpose, shift work, average daily working hours, monthly rest arrangement, perceived occupational meaningfulness, emotional support from colleagues, leadership concern for psychological health, workplace psychological counseling or training opportunities, stress‐reduction methods, family understanding, labor contract status and insurance. Hospital affiliation was recorded directly by the original questionnaire item asking which medical institution each participant came from. The analytical dataset contained a valid hospital entry for all 846 participants from 21 hospitals. Hospital names were converted to internal cluster identifiers for the clustered and multilevel analyses.

#### 2.2.2 Decent Work Perception Scale

The Decent Work Perception Scale was developed by Mao et al. (2014). It consists of 16 items in five dimensions, namely work rewards, work position, career development, professional recognition, and work atmosphere. Each item is scored on a 1–5 scale, with a total score of 16–80, higher scores indicating stronger perception of decent work. Cronbach's alpha in the current study was 0.778.

#### 2.2.3 Occupational Burnout Scale

Occupational burnout was measured using the Chinese version as described by Li and Shi (2003), based on the Maslach framework (Maslach and Jackson 1981, Maslach et al. 2001). The scale had three subscales: emotional exhaustion, depersonalization, and personal accomplishment. The response format is 7‐point (0‐6). Total scores range from 0 to 132 after appropriate direction of the personal accomplishment items, and higher scores indicate higher levels of occupational burnout. Cronbach's alpha for the current study was 0.818.

#### 2.2.4 Psychological Capital Scale

The Chinese questionnaire revised by Luo and He was used to measure the psychological capital (Luo and He 2010). The 24 items measure self‐efficacy, hope, optimism, and resilience and are scored on a 1–6 scale with a total score range of 24 to 144 with higher scores representing higher levels of psychological capital. Cronbach's alpha was 0.898 in this research.

#### 2.2.5 Innovation Climate Scale

Innovation climate was measured with the 12‐item, three‐dimension instrument described and psychometrically evaluated by Shi (2020). The items covered colleague support, supervisor support, and organizational support, with four items per dimension. Items were scored from 1 to 5, giving a total score of 12–60; higher scores indicated a more supportive innovation climate. Cronbach's alpha was 0.909 in the present sample.

### 2.3 Statistical Analysis

Data were analyzed with SPSS 26.0, Python 3.9, and R using clubSandwich 0.7.0; supplementary machine‐learning analyses used scikit‐learn 1.5.2. Continuous variables with normal distribution are expressed as mean and standard deviation, and categorical variables as frequency and percentage. Independent‐samples t tests and one‐way analysis of variance were used for unadjusted comparisons. The primary regression used a data‐driven procedure: variables with two‐tailed univariate *p*<0.05 were considered in model building, and the eight predictors retained in the final model are presented in the results of the primary regression analysis. JD‐R categories were prespecified for theoretical interpretation but were not the statistical selection rule. Education and daily working hours were entered as ordered scores, and binary variables were coded 0 = no and 1 = yes. Monthly rest days were represented by five categories; the comparison yielded F = 1.204 and p = 0.308, so this variable did not enter the primary model. Model assumptions were assessed. The primary linear model was additionally evaluated with shuffled five‐fold cross‐validation using random seed 20260807. Hospital analyses included the ICC, conventional hospital‐cluster robust standard errors, CR2 standard errors with Satterthwaite degrees‐of‐freedom correction, hospital fixed effects, a hospital random‐intercept model, and 3000 hospital‐cluster bootstrap mediation resamples. Full coefficients, standard errors, degrees of freedom, confidence intervals, and *p* values are reported in Table S1.

The exploratory random forest included 31 original predictors: sex, age, marital status, education level, household registration, local birth or long‐term residence, number of children, personality type, daily sleep duration, work unit type, professional rank, employment purpose, labor contract, work experience, daily working hours, shift work, monthly rest days, monthly income, insurance, job type, regular specialty training, occupational meaningfulness, family harmony, colleague emotional support, leadership concern for psychological health, workplace counseling or training, effective stress‐reduction method, family understanding and support, occupational burnout, psychological capital, innovation climate. The primary regression model selected predictors from this candidate pool using univariate screening, whereas this exploratory machine‐learning analysis retained all candidates without *p‐*value‐based exclusion to capture potential non‐linear or interactive effects. Numeric missing values were median‐imputed; categorical missing values were mode‐imputed and one‐hot encoded within each training fold, producing 75 transformed features in the full dataset. Hyperparameters were selected by three‐fold inner cross‐validation within each of five outer folds. The grid comprised 400 or 800 trees; 0.33, 0.67, or 1.0 of transformed features per split (approximately 25, 50, or 75 candidate features); and minimum leaf sizes of 4, 8, or 12. Maximum depth was unrestricted, bootstrap sampling was enabled and squared‐error splitting was used. The final full‐sample tuning selected 800 trees, max_features = 0.33 (approximately 25 of 75 transformed features per split), and minimum leaf size = 4. Predictor importance was calculated on held‐out outer‐fold observations using 30 permutations per fold, providing 150 importance estimates per predictor with mean, SD, and empirical 2.fifth‐97.fifth percentile interval. Because psychological capital and innovation climate were correlated, conditional importance was estimated by permuting each variable within deciles of the other variable in held‐out data. Fold‐specific tuning results are reported in Table S5.

Pearson correlation analysis was used to examine bivariate associations among decent work perception, occupational burnout, psychological capital, and innovation climate. A secondary mediation analysis was performed with PROCESS Model 4 in SPSS 27.0 (Hayes 2018). Psychological capital was specified as the independent variable, occupational burnout as the mediator, and decent work perception as the dependent variable. The model adjusted for leadership concern for employees' psychological health, education level, innovation climate, average daily working hours, workplace psychological counseling or training opportunities, and insurance, which were retained as important contextual covariates from the first‐stage regression model. Bias‐corrected bootstrapping with 5000 resamples was used to estimate 95% confidence intervals for the indirect association; an interval excluding zero was considered statistically significant. In a hospital‐cluster robustness analysis, indirect association was also resampled as clusters of hospitals, to assess whether indirect association would still be detectable with clustered sampling structure. The PROCESS coefficients are reported in their unstandardized form. Since exposure, mediator and outcome were measured simultaneously, the mediation model was interpreted as a theory‐guided statistical indirect association, not as evidence of temporal causality.

## 3 Results

### 3.1 Participant Characteristics

846 medical nursing assistants participated. The sample consisted of 85 men (10.0%) and 761 women (90.0%); of the sample, 804 (95.0%) were married. Among the 846 participants, 7.7% (n = 65) had primary school education or below, 71.0% (n = 601) had junior high school education, and 21.3% (n = 180) had high school education or above. A total of 607 participants (71.7%) reported having a formal labor contract, and 399 (47.2%) reported having purchased insurance.Valid n was 842 for having children and work experience because four observations were missing for each variable; valid *n* was 846 for the other variables shown in Table 1. Several categories were highly imbalanced, including participants without children (*n* = 5), those earning more than 10,000 CNY per month (*n* = 9), and those reporting that their family did not understand their work (*n* = 12); comparisons involving these groups were interpreted cautiously.

**TABLE 1:** Unadjusted comparisons of decent work perception by demographic, work‐related, and organizational variables.

Variable	*N* (%)	Score (mean ± SD)	t/F	*p*
Demographic variables				
Gender				
Male	85(10.0)	51.88 ± 9.54	t = ‐2.014	0.044
Female	761(90.0)	53.89 ± 8.64		
Age (years)			t = ‐0.929	0.353
≤45	221(26.1)	53.22 ± 9.33		
≥46	625(73.9)	53.86 ± 8.53		
Marital status			t = 0.596	0.551
Unmarried/Other	42(5.0)	54.48 ± 10.30		
Married	804(95.0)	53.65 ± 8.67		
Education level			F = 9.943	<0.001
Primary school or below	65(7.7)	56.17 ± 7.98		
Junior high school	601(71.0)	54.21 ± 8.18		
High school or above	180(21.3)	51.07 ± 10.21		
Household registration type			t = ‐0.491	0.623
Agricultural	710(83.9)	53.63 ± 8.67		
Non‐agricultural	136(16.1)	54.03 ± 9.16		
Having children			t = 0.547	0.585
No	5(0.6)	55.80 ± 11.30		
Yes	837(99.4)	53.65 ± 8.74		
Local birth/Long‐term residence			t = 1.324	0.186
No	745(88.1)	53.54 ± 8.81		
Yes	101(11.9)	54.77 ± 8.22		
Average monthly income (CNY)			F = 4.772	0.003
<2500	28(3.3)	49.00 ± 7.86		
2501–5000	435 (51.4%)	53.46 ± 8.65		
5001–10,000	374(44.2)	54.16 ± 8.82		
>10,000	9(1.1)	60.00 ± 7.19		
Personality Type			t = ‐2.216	0.027
Introverted	262(31.0)	52.70 ± 8.56		
Extroverted	584(69.0)	54.14 ± 8.81		
Work‐related variables				
Labor contract			t = ‐3.544	<0.001
No	239(28.3)	52.00 ± 8.89		
Yes	607(71.7)	54.36 ± 8.61		
Professional rank			F = 0.468	0.705
None	86(10.2)	53.16 ± 9.90		
Primary	179(21.2)	53.61 ± 8.83		
Intermediate	243(28.7)	54.22 ± 8.09		
Senior	338(40.0)	53.49 ± 8.88		
Purchased insurance			t = ‐4.417	<0.001
No	447(52.8)	52.45 ± 8.71		
Yes	399(47.2)	55.08 ± 8.60		
Work experience (years)			F = 0.695	0.499
≤10	702(83.0)	53.55 ± 8.67		
11–20	127(15.0)	54.54 ± 8.93		
≥21	13(1.5)	53.38 ± 8.54		
Average daily work hours (h)			F = 11.724	<0.001
≤8	37(4.4)	55.19 ± 9.14		
>8–12	268(31.7)	55.66 ± 8.88		
>12	541(63.9)	52.61 ± 8.48		
Monthly rest days			F = 1.204	0.308
2–4	282(33.3)	54.34 ± 8.98		
5–8	26(3.1)	54.12 ± 6.84		
>8	8(0.9)	55.00 ± 11.78		
Full‐day accompaniment	400(47.3)	53.00 ± 8.67		
Other	130(15.4)	54.25 ± 8.58		
Shift work			t = ‐2.088	0.037
No	282(33.3)	52.80 ± 8.58		
Yes	564(66.7)	54.13 ± 8.81		
Specialized training			F = 1.603	0.187
No regular specialized training	29(3.4)	50.48 ± 7.99		
Provided by employing company	425(50.2)	53.59 ± 8.60		
Provided by medical institution	388(45.9)	54.02 ± 8.97		
Other	4(0.5)	56.00 ± 4.90		
Perceived meaningfulness of occupation			t = ‐3.054	0.002
No	28(3.3)	48.75 ± 9.66		
Yes	818(96.7)	53.86 ± 8.67		
Harmonious family atmosphere			t = ‐0.547	0.585
No	15(1.8)	52.47 ± 9.96		
Yes	831(98.2)	53.71 ± 8.73		
Emotional support from colleagues			t = ‐2.178	0.030
No	67(7.9)	51.46 ± 9.07		
Yes	779(92.1)	53.88 ± 8.70		
Organizational support variables				
Leadership concern for employees' psychological health			t = ‐9.292	<0.001
No	60(7.1)	44.05 ± 7.68		
Yes	786(92.9)	54.43 ± 8.39		
Workplace provides psychological counseling or training opportunities			t = ‐8.571	<0.001
No	111(13.1)	47.32 ± 7.90		
Yes	735(86.9)	54.65 ± 8.47		
Availability of effective stress reduction methods (e.g., Exercise)			t = ‐4.908	<0.001
No	167(19.7)	50.75 ± 8.25		
Yes	679(80.3)	54.41 ± 8.72		
Family understanding of work			t = ‐2.108	0.035
No	12(1.4)	48.42 ± 5.81		
Yes	834(98.6)	53.77 ± 8.76		

*Note*: Valid *n* = 846 unless otherwise indicated. Having children and work experience each had valid *n* = 842 (4 missing). Percentages may not total 100 because of rounding. Comparisons involving very small groups should be interpreted cautiously. *** *p*< 0.001, ** *p*< 0.01, *p < 0.05.

### 3.2 Decent Work Perception

As shown in Table 2, the mean total decent work perception score was 53.69 ± 8.75, and the mean item score was 3.36 ± 0.55. Work atmosphere had the highest item mean (4.01 ± 0.83), whereas work rewards had the lowest (2.90 ± 0.82), indicating comparatively weaker perceptions of compensation and benefits.

**TABLE 2:** Total and dimension scores of decent work perception among medical nursing assistants.

Variable	Score (Mean ± SD)	Item mean score (Mean ± SD)
Work rewards	11.61 ± 3.28	2.90 ± 0.82
Work position	9.86 ± 2.72	3.29 ± 0.91
Career development	10.74 ± 2.06	3.58 ± 0.69
Professional recognition	9.46 ± 3.07	3.15 ± 1.02
Work atmosphere	12.02 ± 2.49	4.01 ± 0.83
Decent work perception (Total)	53.69 ± 8.75	3.36 ± 0.55

### 3.3 Unadjusted Comparisons of Decent Work Perception

In unadjusted analyses, decent work perception scores differed across several demographic, work‐related, and organizational variables (Table 1). These comparisons were descriptive. Monthly rest days comprised five categories, and the five‐category comparison yielded *F* = 1.204, *p* = 0.308, indicating no statistically significant difference across the five groups. Results for very small or highly imbalanced subgroups were interpreted cautiously.

### 3.4 Pearson Correlation Analysis

Pearson correlation analysis showed that decent work perception was negatively correlated with occupational burnout (*r* = ‐0.394, *p*< 0.01) and positively correlated with psychological capital (*r* = 0.524, *p* < 0.01) and innovation climate (*r* = 0.488, *p* < 0.01). Occupational burnout was negatively correlated with psychological capital (*r* = ‐0.353, *p*< 0.01) and innovation climate (*r* = ‐0.319, *p*< 0.01). Psychological capital was positively correlated with innovation climate (*r* = 0.652, *p*< 0.01; Table 3).

**TABLE 3:** Pearson correlation matrix for main study variables (N = 846).

Variable	1	2	3	4
1. Decent work perception	1			
2. Occupational burnout	−0.394**	1		
3. Psychological capital	0.524**	−0.353**	1	
4. Innovation climate	0.488**	−0.319**	0.652**	1

*Note*: Values are Pearson correlation coefficients. Two‐tailed tests were used. **p<0.01.

**TABLE 4:** Multiple linear regression of factors associated with decent work perception among medical nursing assistants (*N* = 846).

Variable	*B*	SE	*β*	*t* Value	*p* Value
Constant	35.122	2.510		13.994	<0.001^***^
Psychological capital	0.142	0.016	0.309	8.749	<0.001^***^
Occupational burnout	−0.098	0.014	−0.194	−6.805	<0.001^***^
Leadership concern for employees' psychological health	3.460	1.014	0.102	3.413	0.001^**^
Education level	−2.537	0.444	−0.151	−5.709	<0.001^***^
Innovation climate	0.179	0.040	0.160	4.448	<0.001^***^
Average daily work hours	−1.464	0.404	−0.096	−3.624	<0.001^***^
Workplace provision of psychological counseling or training	2.641	0.752	0.102	3.513	<0.001^***^
Purchased Insurance	1.517	0.465	0.087	3.262	0.001^**^

*Note: R* = 0.654, R2 = 0.428, adjusted R2 = 0.423, *F* = 78.369, *p*< 0.001. Education level and average daily working hours were entered as ordered scores; binary indicators were coded 0 = no and 1 = yes. *B* = unstandardized coefficient; beta = standardized coefficient; SE = standard error. *** *p*< 0.001, ** *p*< 0.01.

### 3.5 Multiple Linear Regression of Factors Associated With Decent Work Perception

Decent work perception was entered as the dependent variable. Table 4 presents the final results of the primary regression model.The primary regression retained psychological capital, occupational burnout, leadership concern for psychological health, education, innovation climate, daily working hours, workplace counseling or training opportunities, and insurance. Multicollinearity was acceptable (VIF 1.025–1.882; tolerance 0.548–0.975). The model was statistically significant (*F* = 78.369, *p*< 0.001), with R2 = 0.428 and adjusted R2 = 0.423; its five‐fold cross‐validated R2 was 0.414. The hospital ICC was 0.024 across 21 hospitals. With CR2 standard errors and Satterthwaite degrees of freedom, psychological capital (*B* = 0.142, SE = 0.018, df = 3.83, *p* = 0.002), occupational burnout (*B* = ‐0.098, SE = 0.022, df = 4.56, *p* = 0.008), leadership concern (*B* = 3.460, SE = 1.108, df = 3.22, *p* = 0.048), education (*B* = ‐2.537, SE = 0.393, df = 4.07, *p* = 0.003), innovation climate (*B* = 0.179, SE = 0.042, df = 4.20, *p* = 0.012), and counseling or training opportunities (*B* = 2.641, SE = 0.660, df = 2.99, *p* = 0.028) remained statistically significant. Daily working hours (*B* = ‐1.464, SE = 0.531, df = 3.51, *p* = 0.059) and insurance (*B* = 1.517, SE = 0.584, df = 4.27, *p* = 0.057) were attenuated. Table S1 reports complete primary OLS, conventional cluster‐robust, CR2/Satterthwaite, hospital fixed‐effect, and hospital random‐intercept estimates. The random forest nested cross‐validated R2 was 0.393. All five outer‐fold tuning procedures selected max_features = 0.33 (approximately 25 of 75 transformed features per split) and minimum leaf size = 4; the selected number of trees was 400 or 800 across folds. The leading held‐out permutation importances were Psychological capital (mean = 0.138, SD = 0.042, 95% interval 0.064 to 0.217), Occupational burnout (mean = 0.082), and Innovation climate (mean = 0.077). After conditioning on innovation climate, psychological capital retained an importance mean of 0.083 (95% interval 0.014 to 0.183); after conditioning on psychological capital, innovation climate retained an importance mean of 0.033 (95% interval ‐0.009 to 0.080).

### 3.6 Exploratory Statistical Indirect Association Through Occupational Burnout

The mediation model demonstrated that occupational burnout partially mediated the association between psychological capital and decent work perception among medical nursing assistants, as illustrated in Figure 1. The bootstrap 95% confidence interval for the indirect association between psychological capital and decent work perception through occupational burnout did not include zero (indirect association = 0.022, SE = 0.004, 95% CI 0.013–0.032). The direct association was 0.142, and the total association was 0.164. The indirect component represented 13.4% of the total association and the direct component represented 86.6% (Table 5). In the hospital‐cluster bootstrap sensitivity analysis, the indirect association remained statistically detectable (indirect association = 0.022, 95% CI 0.013–0.038, 3,000 cluster bootstrap resamples). This result indicates a statistically detectable indirect association in the specified theory‐driven cross‐sectional model. It does not establish that psychological capital temporally reduces burnout or that burnout causally changes decent work perception.

**TABLE 5:** Decomposition of total, direct, and indirect statistical associations.

Path component	Effect value	SE	LLCI	ULCI	Effect size (%)
Total association	0.164	0.016	0.132	0.196	—
Direct association	0.142	0.016	0.110	0.174	86.6%
Indirect association	0.022	0.004	0.013	0.032	13.4%

**FIGURE 1:**
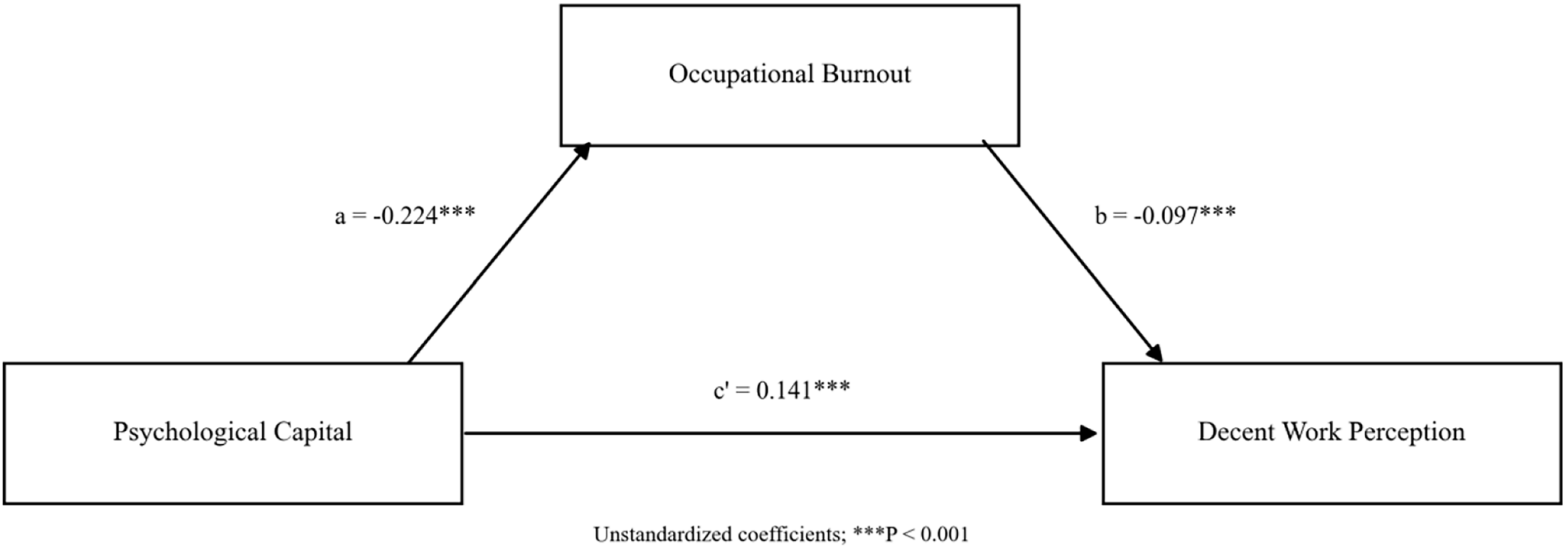
Exploratory mediation model linking psychological capital, occupational burnout, and decent work perception. Values are unstandardized PROCESS coefficients. The model adjusted for leadership concern for employees' psychological health, education level, innovation climate, average daily working hours, workplace psychological counseling or training opportunities, and insurance. *** *p*< 0.001.

## 4 Discussion

This multicenter cross‐sectional study addressed one integrated JD‐R‐guided question. The conceptual roles of demands, resources, and contextual characteristics were defined before interpretation, whereas regression terms were selected using univariate screening. The theoretical categorization and statistical selection therefore served distinct purposes. The linear model and random forest showed comparable out‐of‐sample explanatory performance, with the linear model retaining the advantage of directly interpretable adjusted coefficients. Held‐out and conditional permutation analyses identified psychological capital, occupational burnout, and innovation climate as prominent predictors, although correlated predictors shared information and their rankings were interpreted together rather than as isolated causal contributions.

The indirect association through occupational burnout was 0.022 (95% CI 0.013–0.032), accounting for 13.4% of the total association, and it remained detectable after hospital‐cluster bootstrap resampling. The theoretical ordering of psychological capital, occupational burnout, and decent work perception was selected because it is consistent with three mutually reinforcing perspectives. Psychological capital theory defines psychological capital as a positive personal resource that supports confidence, goal pursuit, optimism, and resilience (Luthans and Youssef‐Morgan 2017). Conservation of resources theory suggests that individuals with greater resources are less vulnerable to stress‐induced resource loss (Hobfoll 1989). The JD‐R health‐impairment pathway further proposes that demands and resources shape burnout, which in turn influences work‐related psychological outcomes (Bakker and Demerouti 2017). Under this integrated view, psychological capital is positioned as an antecedent personal resource, occupational burnout as a resource‐depletion state, and decent work perception as a downstream appraisal of whether work is secure, rewarding, dignified, and developmentally meaningful. Alternative or reciprocal directions remain possible, especially because all variables were measured at one time point, and Ferraro et al. treated decent work as a predictor of psychological capital (Ferraro et al. 2018). We therefore do not claim causal mediation. Rather, we selected the current ordering as the main model because it best matches the resource‐protection and health‐impairment question addressed by this study.

Higher education category and longer daily working hours were negatively associated with decent work perception. A possible explanation for the education finding is person‐job mismatch: workers with more education may have stronger expectations for development, recognition, and rewards that are not fully met by auxiliary care roles (Khan et al. 2022, Liu et al. 2025). Longer working hours may reflect greater job demands and poorer work‐life balance. A supportive innovation climate was positively associated with decent work perception and may reflect greater opportunities for learning, participation, and recognition (Shi 2020).

Leadership concern for psychological health, workplace counseling or training opportunities, and insurance were positively associated with decent work perception. These findings may indicate the relevance of organizational support, occupational security, organizational belongingness, and perceived work stability rather than any unmeasured social‐status benefit. Supportive management and social support have been linked with more favorable psychological and work‐related outcomes among healthcare workers (Gui et al. 2025, Xia et al. 2025, Zhao et al. 2025). Because the present study was observational, these associations should inform hypotheses and service planning rather than be interpreted as proven intervention effects.

The findings suggest several practical directions that merit prospective evaluation. Medical institutions could assess work‐hour arrangements, access to insurance, leadership support, psychological services, training opportunities, and innovation climate. Programs designed to strengthen psychological capital or reduce occupational burnout should be tested before claims of effectiveness are made. Future studies of no‐accompanying care models should directly measure workload, staffing, compensation, shift patterns, occupational burnout, and decent work perception rather than assuming that the model necessarily increases burnout or reduces mental health.

The study was conducted on a large sample from 21 general hospitals and studied an under‐studied auxiliary workforce. However, important limitations still remain. The sample is limited by convenience sampling within one province and thus not highly representative, limiting generalizability. All core variables were self‐reported at a single time point, and this may introduce common method bias (Podsakoff et al. 2003). Item‐level observations were unavailable for a single‐factor confirmatory factor analysis or an unmeasured method‐factor model. The primary regression used univariate screening at*p*<0.05, so the selected terms may be sample‐dependent and theoretically relevant variables may have been excluded. Nested cross‐validation and complete random‐forest importance results quantified out‐of‐sample performance and ranking uncertainty but did not convert the screening procedure into a prespecified model. Hospital cluster sizes were markedly imbalanced (minimum 1, median 7, maximum 270 participants). CR2 standard errors with Satterthwaite degrees of freedom addressed the limited number of clusters, but the very small effective degrees of freedom for some coefficients reduced precision; daily working hours and insurance did not remain statistically significant under this correction. Four variables were missing for having children and work experience and several categories were very small or very unbalanced. Cross‐sectional mediation does not demonstrate temporal order, and other or reciprocal directions are plausible. Therefore, the mediation result should be treated as a theory‐guided exploratory statistical indirect association only.

## 5 Conclusion

Perception of decent work among Chinese medical nursing assistants was statistically associated with psychological capital, occupational burnout, daily working hours, education, innovation climate, leadership concern for psychological health, workplace counseling or training opportunities, and insurance. Hospital‐level models, five‐fold linear‐model validation, nested random‐forest validation, and repeated held‐out permutation analyses showed that the main associations and leading predictors were stable across complementary analyses. Occupational burnout was statistically associated with a small proportion (13.4%) of the cross‐sectional association between psychological capital and decent work perception. The model ordering is supported by JD‐R, conservation of resources, and psychological capital theory, but the results do not prove causal mediation or intervention effectiveness. Management recommendations need to be evidence‐based and therefore longitudinal, intervention, and hospital‐cluster‐aware studies are needed.

## Author Contributions

**Jinhong Lu**: conceptualization, data curation, formal analysis, investigation, methodology, resources, supervision, validation, visualization, writing – original draft, writing – review and editing. **Juan Liu**: conceptualization, investigation, writing – original draft, methodology, validation, visualization, writing – review and editing, formal analysis, data curation, supervision, resources. **Huina Mao**: project administration, data curation, investigation, supervision. **Guizhi Luo**: software, resources.

## Funding

This study was supported by the Guangdong Medical Science and Technology Research Fund (Grant No. A2026109) and the Dean’s Research Fund of Zhujiang Hospital, Southern Medical Universit (Grant No. yzjj2025hlsj04).

## Conflicts of Interest

The authors declare no conflicts of interest.

## Ethics Statement

This study has been reviewed by the ethics committee of the hospital (ethics number: 2025‐KY‐478‐01) and follows ethical principles.

## Supporting information




**Supplementary Tables**: brb371752‐sup‐0001‐TableS1‐S5.docx

## Data Availability

The datasets generated and/or analyzed during the current study are not publicly available due to patient privacy and ethical restrictions but are available from the corresponding author on reasonable request and with approval from the institutional ethics committee (No. 2025‐KY‐478‐01).
