# Supplementary material for: Factors Associated With Decent Work Perception Among Chinese Medical Nursing Assistants: A Cross‐Sectional Study With Exploratory Mediation Analysis
Source: Brain Behav. 2026 Oct 13;16(10):e71752. doi: 10.1002/brb3.71752 (PMC13659933; doi:10.1002/brb3.71752)
Supplement: Supplementary file 1 [file BRB3-16-e71752-s001.docx]

**Supplementary Table 1. Full coefficient estimates from primary and hospital-level robustness models**

| **Model** | **Variable** | **B** | **SE** | **df** | **95% CI** | **P** |
| --- | --- | --- | --- | --- | --- | --- |
| Primary OLS | Psychological capital | 0.142 | 0.016 |  | 0.110 to 0.174 | 1.18e-17 |
| Primary OLS | Occupational burnout | -0.098 | 0.014 |  | -0.126 to -0.070 | 1.93e-11 |
| Primary OLS | Leadership concern | 3.460 | 1.014 |  | 1.470 to 5.449 | 6.73e-04 |
| Primary OLS | Education level | -2.537 | 0.444 |  | -3.409 to -1.665 | 1.57e-08 |
| Primary OLS | Innovation climate | 0.179 | 0.040 |  | 0.100 to 0.258 | 9.84e-06 |
| Primary OLS | Daily working hours | -1.464 | 0.404 |  | -2.257 to -0.671 | 3.07e-04 |
| Primary OLS | Counseling or training | 2.641 | 0.752 |  | 1.165 to 4.117 | 4.68e-04 |
| Primary OLS | Insurance | 1.517 | 0.465 |  | 0.604 to 2.430 | 0.001 |
| Cluster robust | Psychological capital | 0.142 | 0.016 |  | 0.108 to 0.175 | 2.49e-08 |
| Cluster robust | Occupational burnout | -0.098 | 0.020 |  | -0.139 to -0.057 | 7.49e-05 |
| Cluster robust | Leadership concern | 3.460 | 0.989 |  | 1.397 to 5.522 | 0.002 |
| Cluster robust | Education level | -2.537 | 0.380 |  | -3.330 to -1.745 | 1.68e-06 |
| Cluster robust | Innovation climate | 0.179 | 0.039 |  | 0.098 to 0.260 | 1.69e-04 |
| Cluster robust | Daily working hours | -1.464 | 0.466 |  | -2.437 to -0.492 | 0.005 |
| Cluster robust | Counseling or training | 2.641 | 0.629 |  | 1.329 to 3.953 | 4.42e-04 |
| Cluster robust | Insurance | 1.517 | 0.548 |  | 0.374 to 2.660 | 0.012 |
| CR2/Satt. | Psychological capital | 0.142 | 0.018 | 3.83 | 0.090 to 0.194 | 0.002 |
| CR2/Satt. | Occupational burnout | -0.098 | 0.022 | 4.56 | -0.155 to -0.040 | 0.008 |
| CR2/Satt. | Leadership concern | 3.460 | 1.108 | 3.22 | 0.066 to 6.853 | 0.048 |
| CR2/Satt. | Education level | -2.537 | 0.393 | 4.07 | -3.622 to -1.453 | 0.003 |
| CR2/Satt. | Innovation climate | 0.179 | 0.042 | 4.20 | 0.064 to 0.294 | 0.012 |
| CR2/Satt. | Daily working hours | -1.464 | 0.531 | 3.51 | -3.023 to 0.094 | 0.059 |
| CR2/Satt. | Counseling or training | 2.641 | 0.660 | 2.99 | 0.534 to 4.748 | 0.028 |
| CR2/Satt. | Insurance | 1.517 | 0.584 | 4.27 | -0.066 to 3.100 | 0.057 |
| Hospital FE | Psychological capital | 0.136 | 0.016 |  | 0.104 to 0.169 | 4.23e-16 |
| Hospital FE | Occupational burnout | -0.099 | 0.015 |  | -0.129 to -0.070 | 5.16e-11 |
| Hospital FE | Leadership concern | 3.294 | 1.022 |  | 1.289 to 5.299 | 0.001 |
| Hospital FE | Education level | -2.535 | 0.462 |  | -3.442 to -1.628 | 5.40e-08 |
| Hospital FE | Innovation climate | 0.184 | 0.041 |  | 0.105 to 0.264 | 6.64e-06 |
| Hospital FE | Daily working hours | -1.590 | 0.420 |  | -2.414 to -0.767 | 1.61e-04 |
| Hospital FE | Counseling or training | 2.560 | 0.759 |  | 1.070 to 4.050 | 7.80e-04 |
| Hospital FE | Insurance | 1.491 | 0.477 |  | 0.555 to 2.427 | 0.002 |
| Random intercept | Psychological capital | 0.140 | 0.016 |  | 0.109 to 0.172 | 3.67e-18 |
| Random intercept | Occupational burnout | -0.097 | 0.014 |  | -0.125 to -0.069 | 1.20e-11 |
| Random intercept | Leadership concern | 3.384 | 1.010 |  | 1.404 to 5.363 | 8.07e-04 |
| Random intercept | Education level | -2.532 | 0.443 |  | -3.401 to -1.663 | 1.12e-08 |
| Random intercept | Innovation climate | 0.180 | 0.040 |  | 0.102 to 0.259 | 6.78e-06 |
| Random intercept | Daily working hours | -1.542 | 0.411 |  | -2.347 to -0.736 | 1.76e-04 |
| Random intercept | Counseling or training | 2.618 | 0.749 |  | 1.151 to 4.085 | 4.70e-04 |
| Random intercept | Insurance | 1.503 | 0.463 |  | 0.595 to 2.411 | 0.001 |

**Supplementary Table 2. Held-out permutation importance (decrease in R2) for all random forest predictors**

| **Rank** | **Variable** | **Importance mean** | **Importance SD** | **95% interval** | **Positive repeats (%)** |
| --- | --- | --- | --- | --- | --- |
| 1 | Psychological capital | 0.138 | 0.042 | 0.064 to 0.217 | 100.000 |
| 2 | Occupational burnout | 0.082 | 0.025 | 0.040 to 0.126 | 100.000 |
| 3 | Innovation climate | 0.077 | 0.031 | 0.016 to 0.131 | 100.000 |
| 4 | Leadership concern for psychological health | 0.028 | 0.018 | -0.003 to 0.063 | 96.000 |
| 5 | Workplace counseling or training | 0.019 | 0.011 | -0.005 to 0.038 | 93.333 |
| 6 | Education level | 0.011 | 0.009 | -0.010 to 0.025 | 86.667 |
| 7 | Insurance | 0.007 | 0.007 | -0.011 to 0.020 | 88.000 |
| 8 | Daily working hours | 0.006 | 0.009 | -0.014 to 0.020 | 83.333 |
| 9 | Daily sleep duration | 0.006 | 0.006 | -0.007 to 0.015 | 85.333 |
| 10 | Monthly rest days | 0.005 | 0.005 | -0.004 to 0.014 | 82.667 |
| 11 | Work experience | 0.004 | 0.005 | -0.004 to 0.013 | 81.333 |
| 12 | Sex | 0.002 | 0.002 | -0.001 to 0.005 | 87.333 |
| 13 | Professional rank | 0.001 | 0.004 | -0.007 to 0.008 | 64.667 |
| 14 | Personality type | 0.001 | 0.004 | -0.007 to 0.007 | 64.000 |
| 15 | Household registration | 0.001 | 0.002 | -0.003 to 0.007 | 70.667 |
| 16 | Employment purpose | 9.83e-04 | 0.006 | -0.010 to 0.012 | 60.000 |
| 17 | Effective stress-reduction method | 3.23e-04 | 0.002 | -0.005 to 0.004 | 58.667 |
| 18 | Number of children | 1.92e-04 | 0.002 | -0.003 to 0.004 | 54.000 |
| 19 | Shift work | 1.89e-04 | 0.003 | -0.005 to 0.004 | 58.000 |
| 20 | Job type | 1.89e-04 | 0.001 | -0.003 to 0.002 | 69.333 |
| 21 | Labor contract | 8.90e-05 | 0.002 | -0.004 to 0.004 | 56.000 |
| 22 | Age | 1.95e-05 | 0.004 | -0.008 to 0.007 | 52.667 |
| 23 | Family harmony | 1.03e-05 | 1.33e-04 | -0.000 to 0.000 | 37.333 |
| 24 | Family understanding and support | 3.57e-06 | 3.72e-05 | -0.000 to 0.000 | 19.333 |
| 25 | Occupational meaningfulness | -1.17e-04 | 4.42e-04 | -0.001 to 0.001 | 46.667 |
| 26 | Marital status | -1.62e-04 | 3.68e-04 | -0.001 to 0.001 | 28.000 |
| 27 | Colleague emotional support | -1.75e-04 | 0.001 | -0.002 to 0.002 | 51.333 |
| 28 | Local birth or long-term residence | -5.40e-04 | 7.72e-04 | -0.003 to 0.001 | 20.000 |
| 29 | Monthly income | -5.84e-04 | 0.005 | -0.012 to 0.006 | 51.333 |
| 30 | Regular specialty training | -6.27e-04 | 0.004 | -0.011 to 0.006 | 46.667 |
| 31 | Work unit type | -0.002 | 0.004 | -0.010 to 0.003 | 36.000 |

**Supplementary Table 3. Conditional permutation importance (decrease in R2) for correlated predictors**

| **Variable** | **Conditioned on** | **Importance mean** | **Importance SD** | **95% interval** | **Positive repeats (%)** |
| --- | --- | --- | --- | --- | --- |
| Psychological capital | Innovation climate | 0.083 | 0.048 | 0.014 to 0.183 | 99.500 |
| Innovation climate | Psychological capital | 0.033 | 0.023 | -0.009 to 0.080 | 95.000 |

**Supplementary Table 4. Complete predictor list for the random forest analysis**

| **Predictor** | **Data type** |
| --- | --- |
| Sex | Categorical |
| Age | Numeric |
| Marital status | Categorical |
| Education level | Categorical |
| Household registration | Categorical |
| Local birth or long-term residence | Categorical |
| Number of children | Numeric |
| Personality type | Categorical |
| Daily sleep duration | Numeric |
| Work unit type | Categorical |
| Professional rank | Categorical |
| Employment purpose | Categorical |
| Labor contract | Categorical |
| Work experience | Numeric |
| Daily working hours | Categorical |
| Shift work | Categorical |
| Monthly rest days | Categorical |
| Monthly income | Categorical |
| Insurance | Categorical |
| Job type | Categorical |
| Regular specialty training | Categorical |
| Occupational meaningfulness | Categorical |
| Family harmony | Categorical |
| Colleague emotional support | Categorical |
| Leadership concern for psychological health | Categorical |
| Workplace counseling or training | Categorical |
| Effective stress-reduction method | Categorical |
| Family understanding and support | Categorical |
| Occupational burnout | Numeric |
| Psychological capital | Numeric |
| Innovation climate | Numeric |

**Supplementary Table 5. Nested cross-validation tuning results**

| **Stage** | **Inner-CV best R2** | **Trees** | **Candidate features per split** | **Minimum leaf size** |
| --- | --- | --- | --- | --- |
| Outer fold 1 | 0.395 | 800 | 25 (0.33) | 4 |
| Outer fold 2 | 0.366 | 400 | 25 (0.33) | 4 |
| Outer fold 3 | 0.401 | 400 | 25 (0.33) | 4 |
| Outer fold 4 | 0.372 | 400 | 25 (0.33) | 4 |
| Outer fold 5 | 0.404 | 800 | 25 (0.33) | 4 |
| Final full-sample fit |  | 800 | 25 (0.33) | 4 |
